# Case Report: Hyperplastic Callus of the Femur Mimicking Osteosarcoma in Osteogenesis Imperfecta Type V

**DOI:** 10.3389/fendo.2021.622674

**Published:** 2021-04-15

**Authors:** Ying Deng, Yanan Huo, Jinfeng Li

**Affiliations:** Department of Endocrine, Jiangxi Provincial People’s Hospital Affiliated to Nanchang University, Nanchang, China

**Keywords:** osteogenesis imperfecta, type V, bony callus, pathology, osteosarcoma

## Abstract

**Background:**

Osteogenesis imperfecta (OI) type V is a rare form of OI which is often characterized by hyperplastic callus. Misdiagnosis is a possibility due to its rarity and because patients involved are mostly in adolescence, a predisposing age for osteosarcoma. Here, we report this case and aim to improve understanding of patients with OI type V and avoid misdiagnosis.

**Case Presentation:**

A male, 14-year-old patient was admitted to Jiangxi Provincial People’s Hospital affiliated to Nanchang University in August 2020 due to repeated fractures for more than 11 years and swelling in his right leg for more than 4 years. The patient was diagnosed with OI in 2014 due to repeated fracture and was treated with bisphosphonates. The swelling was accompanied by huge callus formation. Prior to admission to our hospital in 2016 osteosarcoma was suspected by imaging and pathology, and amputation was recommended. OI-V was confirmed after more than four years of follow-up and genetic diagnosis, and the affected limb was preserved.

**Conclusion:**

The history of OI and lack of rapid progression suggested OI-V with a hyperplastic callus. Combined with genetic testing, the diagnosis was OI-V. Although the patient was at a predisposing age for osteosarcoma, diagnosis and treatment should be based on the medical history of the patient, imaging,and genetic testing, and sometimes even time-consuming retrospective observation.

## Introduction

Osteogenesis imperfecta (OI), a monogenic hereditary disease involving bone and connective tissue, also referred to as fragile bone disease. It is characterized by repeated fractures, blue sclera, enamel dysplasia, and hearing loss and is currently classified into 15 types (1, 2). More than 90% result from mutations in the gene for type I collagen-coding protein (COL1A1, COL1A2). OI type V (OI-V) is a rare form caused by mutation of the gene for interferon-induced transmembrane protein 5 (IFITM5) and shows autosomal dominant inheritance (3–6). In this study, we report a case diagnosed with OI and suspected osteosarcoma, which was followed up for more than 4 years, and the suspected osteosarcoma was eventually confirmed to be OI-V by genetic analysis.

## Case Description

A male 14 years, Han nationality, was admitted again to Jiangxi Provincial People’s Hospital affiliated to Nanchang University in August 2020 due to repeated fractures for more than 11 years and swelling in his right leg for more than 4 years. The case was born of a parity 1, gravida 1; mother and the parents were not close relatives. Granny had lung cancer; father died of glioma and had dislocation of radius head, with no history of fracture. There was no family history of fracture and blue sclera.

At the age of three, a left radius fracture occurred after falling on flat ground and healed spontaneously after plaster fixation. Later, left clavicle, bilateral tibiofibular fractures and dislocation of left radial head occurred successively due to falls. In 2013, a right femoral shaft fracture was caused by a tumble, and plate internal fixation was performed. In February 2014, the patient was admitted to Jiangxi Provincial People’s Hospital affiliated to Nanchang University; physical examination showed unequal legs, scoliosis, dislocation of left radial head, slightly loose ligaments, combined with repeatedly brittle fractures, and low bone density. He was diagnosed as “Osteogenesis Imperfecta” and was treated with pamidronate disodium: 15 mg on 18 February, 2014; 30 mg on 29th May, 2014; 15 mg on 29th August, 2014; 30 mg on 4th February, 2015, accompanied by calcium and vitamins A and D (The patient did not purchase vitamin D and used the vitamin AD mixture). Calcium and vitamins A and D were occasionally missed but no fracture occurred. In July 2016, the treatment was changed to intravenous zoledronic acid 5 mg (5 mg is an annual dose) and after that the patient went back home. In August 2016, without obvious inducement, pain and swelling in the middle and lower parts of the right thigh occurred, and local skin temperature increased and slowly aggravated.

**Figure 1:**
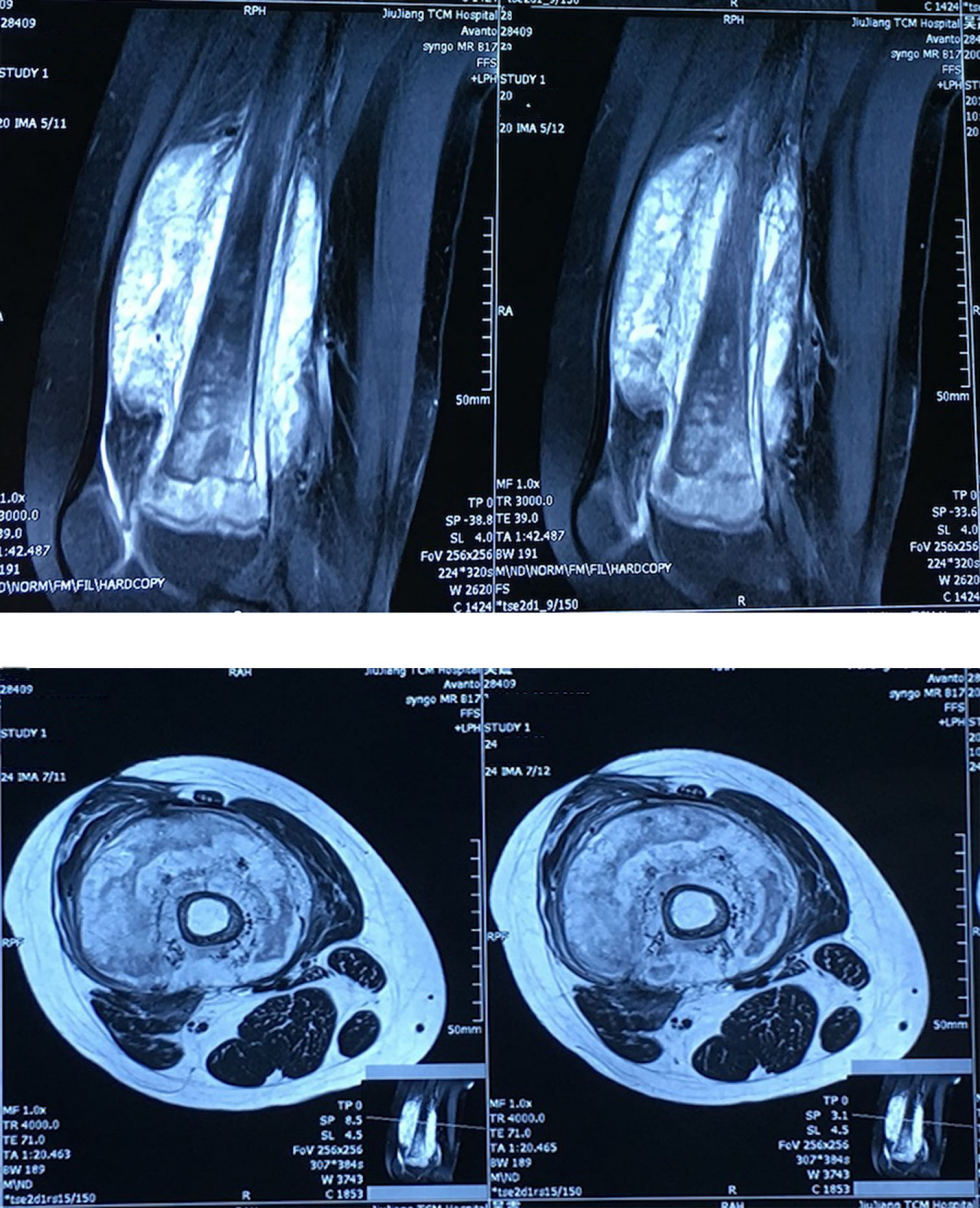
Enhanced magnetic resonance imaging (MRI) of the right thigh revealed bone thinning of the right femur, surrounding a soft tissue mass shadow around the femur and high possibility of malignant tumor.

X-ray of the right femur in Duchang Hospital of Traditional Chinese Medicine in October 2016 showed swelling of the right femur, slightly thickened bone texture, thin bone cortex, and no periosteal reaction; enhanced magnetic resonance imaging (MRI) at Jiujiang Hospital of Traditional Chinese Medicine of the right thigh revealed bone thinning of the right femur, surrounding a soft tissue mass shadow around the femur and high possibility of malignant tumor (**Figure 1**).

On October 28, 2016, the patient was admitted to Jiujiang Hospital of Traditional Chinese Medicine and underwent biopsy of the lesions in the middle and lower parts of the thigh. The pathology indicated periosteal osteosarcoma for the lateral mass at the mid-low segment of the right leg. However, the possibility of myositis ossificans could not be ruled out. Without exception of malignancy amputation was recommended.

Pathological result from Beijing Jishuitan Hospital in November 2016 showed edematous fibrous tissue with new spindle cells, a small number of small lymphocytes, a large amount of local bone-like and cartilage-like matrix (partly composed of trabeculae, lined by osteoblasts), and a small number of osteoblasts between nearby bone (part atrophy) and adipocytes. Pathology suggested periosteal osteosarcoma. But the imaging series over 3 months suggested heterotopic ossification of the extra-skeletal soft tissues (myositis ossificans) on 10th November, 2016. On 11th November, 2016, pathological result from the People’s Hospital of Peking University showed a huge mass encapsulating the femur and the possibility of periosteal osteosarcoma. However, considering the rapid disease progression, the clinical history of fracture and medication history for osteogenesis imperfecta, the possibility of a giant abnormal osteophyte formation or myositis ossificans accompanied with pathological changes were also considered. The diagnosis was still indefinite and close follow-up was suggested.

The patient was admitted to Jiangxi Provincial People’s Hospital affiliated to Nanchang University again in August 2017. The pain gradually eased, and the swelling of the right affected limb was alleviated.

**Figure 2:**
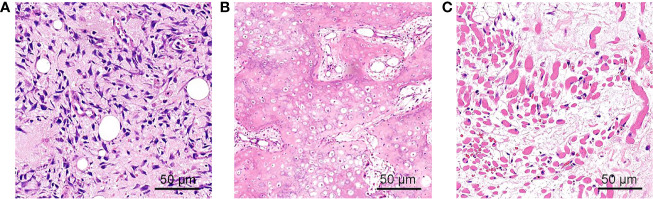
The HE staining of pathological specimens. **(A)** Neoplastic spindle cells and edema interstitial; **(B)** cartilage-like matrix and bone-like matrix with osteoblast cells lined around. **(C)** Atrophic striated muscle tissue.

Blood samples were collected (**Table 1**), and the patient was preliminarily diagnosed as OI-V. With the consent of relatives, peripheral blood was taken for gene detection. A missense mutation (heterozygous mutation) of 17A129-1 at 5′UTR was found, resulting in p. M1TextM-5. IFITM5 mutation (c, -14C >T) (**Supplementary Figure 1**). HE staining was also performed on previous pathological specimens (**Figure 2**).

**Table 1:** The bone metabolic assessment of the patient during his admissions.

	Ca	P	ALP	Vit-D	β-CTX	PINP	PTH
2014.2	2.43	1.79	400	6.70	1.24	437.3	25.16
2015.7	2.60	1.69	353	21.31	1.27	390.8	20.28
2016.7	2.28	1.69	464	26.44	1.58	527.5	—
2017.8	2.33	1.38	446	13.35	0.97	349.9	36.68
2019.8	2.31	1.66	473	15.32	1.47	608.2	60.67
2020.8	2.30	1.47	273	11.80	1.23	354.6	52.26

ALP, alkaline phosphatase; TPINP, total N-terminal propeptide of type I procollagen; β-CTX, β-C-terminal telopeptide of type I collagen.

After admission, bone metabolic assessment (**Table 1**) and bone mineral density (**Supplementary Table 1**) were supplemented; part of long bone X-rays were reexamined; a lateral radiograph of the thoracolumbar spine showed multiple fractures. Intravenous zoledronic acid 5 mg, calcium carbonate (500 mg once a day containing calcium), and vitamin D drops 400 IU once to twice a day (occasionally missed) were continued with close observation. The pain gradually disappeared, and the swelling of the right affected limb was alleviated.

The patient was admitted again to the Jiangxi Provincial People’s Hospital affiliated to Nanchang University in July 2018 and in August 2019. Bone metabolic assessment (**Table 1**) and bone mineral density (**Supplementary Table 1**) were supplemented. The patient was treated with Zoledronic acid 5 mg per year—both in 2018 and 2019.

**Figure 3:**
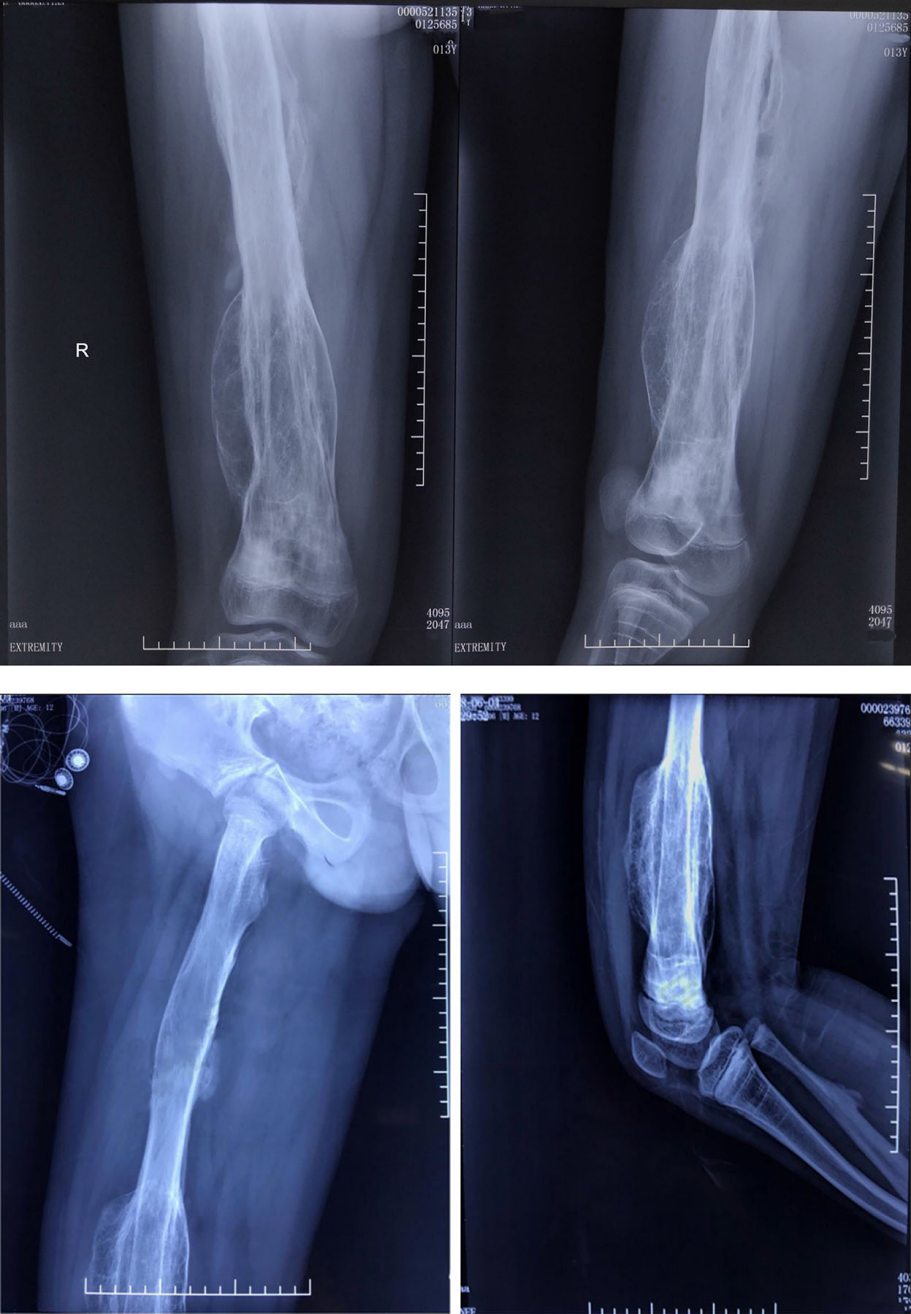
X-ray of the right femur indicated inhomogeneous bone density losses of the right femur, cortical thinning, transverse sclerotic lines at the right distal femur and interface of the epiphysis and metaphysis of the proximal tibia, and excessive callus formation at the cortical margins.

As of 23rd August 2020, the patient was admitted again. There was no redness and slight-swelling in the right thigh; however, the right leg was still slightly thicker than the left leg (left: 43cm right: 45cm) (**Supplementary Table 2**). The patient had no more fractures, the local skin temperature was normal, the condition was stable, and amputation was avoided. Biochemical parameters are shown-in **Table 1**. An X-ray of the right femur indicated inhomogeneous bone density losses of the right femur, cortical thinning, transverse sclerotic lines at the right distal femur and interface of the epiphysis and metaphysis of the proximal tibia, and excessive callus formation at the cortical margins. The callus was smaller than in 2016 (**Figure 3**). In conclusion, the findings suggested OI-V with hyperplastic callus. However, considering 6 years administration of bisphosphonate, we did not treat the patient with bisphosphonate at that time.

## Diagnostic Assessment

This case, with repeated brittle fractures, slightly loose ligaments, scoliosis, and bone density lower than their peers although there was no blue sclera and no hearing loss was preliminarily considered for osteogenesis. imperfect diagnose, and considered for osteogenesis imperfecta. The patient was administered with calcium and vitamin D (Pamidronate first two years and then followed Zoledronic acid for four years), during which time there were no new fractures. Osteosarcoma was suspected by imaging and pathology when his right thigh suffered swelling and pain without obvious inducement, and local skin temperature increased and slowly aggravated, and amputation was recommended. However, the dislocation of his left radial head combined with the images, pathological examination, and genetic testing, excluded-osteosarcoma, and OI-V with hyperplastic callus was confirmed. The callus gradually narrowed, and the patient has not suffered any more fractures, which also support this diagnosis.

## Discussion

In this case, the patient had hyperplastic callus (HPC) and dislocation of the left radial head but no calcification of the forearm interosseous membrane, no hearing loss, no blue sclera. In a disease with such obvious heterogeneity, a diagnosis based on clinical manifestations is obviously inaccurate. This highlights the limitations of the management of this case, which represents an example of misdiagnosis that could have had serious implications for the patient, as he was recommended to undergo amputation for osteosarcoma. Fortunately, when admitted to our hospital a review of the imaging and clinical data, new data and genetic testing all led to an accurate diagnosis. This emphasizes the need for knowledge of OI-V. When we treat patients who suffered HPC especially adolescents, we should first pay attention to x-ray, whether there is a periosteum reaction and calcification of the forearm interosseous membrane; second, it is important to note any history of recurrent fractures and whether there is a family history; third, whether the patient has blue sclera, dislocation of radial head, loose ligaments and hearing loss, and last but not least, DXA and OI genetic testing should be taken into consideration in order to make the correct diagnosis.

The Chinese Union Medical College Hospital reported five cases of OI-V type patients with IFITM5 gene mutation, including femoral HPC, no blue sclera, and enamel dysplasia. The clinical phenotypes of each patient were significantly different including two patients from one family with different clinical phenotypes (7). International studies also show clinical phenotypes differed extremely. In Brazil, among the seven OI-V patients with IFITM5 gene mutation, three had HPC, two had blue sclera and no enamel dysplasia (8).

The incidence of HPC in OI-V is as high as 64%. Therefore, the patient was considered to be OI-V which was confirmed as IFITM5 mutation later. There are reports of OI with osteosarcoma (9). Therefore, we still need to follow up this patient. After a period of rapid growth, the shape and the size of HPC remain unchanged for years (10); some diaphysis will return to normal form, and some of the callus can be left to varying degrees.

Randomized controlled trials of bisphosphonates in OI have consistently shown that BMD is increased, but the effects of bisphosphonates on fracture rate are uncertain (11). As to our patient, he had no more fractures after 6 years’ administration of bisphosphonate, and his bone density increased, his Z-score was above −2.0; meanwhile, considering the drug holiday of bisphosphonates, we didn’t administrate bisphosphonates at the latest admission. However, we are going to observe the clinical symptoms, changes in biochemical parameters, and BMD of the patient so as to timely adjust his treatment.

In conclusion, the patient in this case was confirmed as a rare case of OI-V by genetic analysis. According to their history and current recovery, OI-V combined with HPC is the current diagnosis, but follow-up is needed.

## Data Availability Statement

The original contributions presented in the study are included in the article/**Supplementary Material**. Further inquiries can be directed to the corresponding author.

## Ethics Statement 

The studies involving human participants were reviewed and approved by the Jiangxi Provincial People’s Hospital Affiliated to Nanchang University. Written informed consent to participate in this study was provided by the participants’ legal guardian/next of kin.

## Author Contributions

JL collected the history of the patient. YD designed and wrote the manuscript. YH made manuscript revisions. All authors contributed to the article and approved the submitted version.

## Funding

This study is supported by the project of “metabolic disease specific disease cohort study” (Project No: 2016yfc0901200), the key special project of “precision medicine” of National Key Research and Development Plan in 2016 (Project No: 2016yfc0901205, project name: establishment and sharing of monitoring system for large-scale metabolic disease cohort and new events).

## Conflict of Interest

The authors declare that the research was conducted in the absence of any commercial or financial relationships that could be construed as a potential conflict of interest.
