# Supplementary material for: Case Report: Hyperplastic Callus of the Femur Mimicking Osteosarcoma in Osteogenesis Imperfecta Type V
Source: Front Endocrinol (Lausanne). 2021 Apr 15;12:622674. doi: 10.3389/fendo.2021.622674 (PMC8082416; doi:10.3389/fendo.2021.622674)
Supplement: Supplementary file 1 [file DataSheet_1.doc]

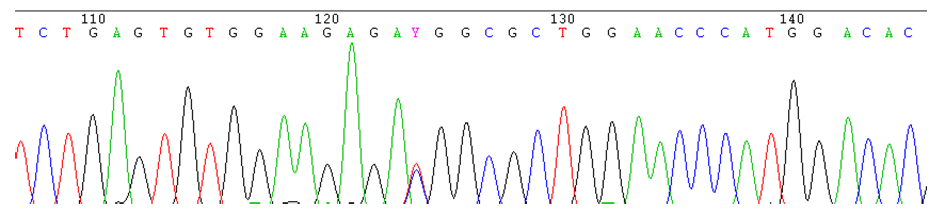


**Supplementary Figure 1**. Map showing identification of the IFITM5 gene mutation in this case of a 14-year-old male with osteogenesis imperfecta. Genetic testing indicated a missense mutation (heterozygous mutation) in the 5’UTR of 17A129-1, leading to p.M1TextM-5. IFITM5 mutation (c,-14C > T).

**Supplementary Table 1.** The DXA assessment of the patient during his admissions.

|  | Fem Neck | | Total | | L2-L4/L1-L4 | | Left forearm | |
| --- | --- | --- | --- | --- | --- | --- | --- | --- |
|  | BMD | T/Z | BMD | T/Z | BMD | T/Z | BMD | Z |
| 2014.2.18 | 0.6805 | -3.12 | 0.6755 | -3.45 | 0.3325 | -4.13 | — | — |
| 2014.8.28 | 0.5464 | -4.26 | 0.6267 | -3.87 | 0.4111 | -3.64 | — | — |
| 2016.7.25 | 0.6906 | -3.03 | 0.7329 | -2.95 | 0.4132 | -3.63 | — | — |
| 2017.8.14 | 0.7379 | -2.63 | 0.7459 | -2.84 | 0.5380 | -2.85 | — | — |
| 2018.6.4 | 0.682 | -1.1 | 0.757 | -0.8 | 0.619 | -0.9 | 0.497 | -1.5 |
| 2019.8.15 | 0.747 | -0.8 | 0.829 | -0.6 | 0.701 | -0.7 | 0.507 | -2.0 |
| 2020.8.24 | 0.814 | -0.6 | 0.910 | -0.3 | 0.808 | -0.3 | — | — |

In 2014-2017 we used Norland, and we just measured T-score, as we didn’t have the reference range of adolescents.

In 2018-2020 we changed to Hologic, and measuered the Z-score.

**Supplementary Table 2.** The auxological parameters of the patient during his admissions.

|  | Height | Weight | Left lower limb length | Right lower limb length | Left thigh circumference | Right thigh circumference |
| --- | --- | --- | --- | --- | --- | --- |
| 2014.2.18 | 129 | 27 | — | — | — | — |
| 2014.8.28 | 129 | 27 | — | — | — | — |
| 2016.7.25 | 134 | 41 | — | — | — | — |
| 2017.8.14 | 136 | 45 | 79 | 80 | 44 | 47 |
| 2018.6.4 | 144 | 50 | 83 | 84 | 44 | 47 |
| 2019.8.15 | 154 | 53 | — | — | — | — |
| 2020.8.24 | 158 | 63 | 91 | 93 | 43 | 45 |
